# Factors associated with girl child marriage in Iran: a qualitative socio-ecological approach

**DOI:** 10.3389/fpubh.2024.1477197

**Published:** 2025-01-21

**Authors:** Asma Pourtaheri, Mehrsadat Mahdizadeh, Hadi Tehrani, Jamshid Jamali, Nooshin Peyman

**Affiliations:** ^1^Health Education and Health Promotion, Student Research Committee, Mashhad University of Medical Sciences, Mashhad, Iran; ^2^Department of Health Education and Health Promotion, School of Health, Mashhad University of Medical Sciences, Mashhad, Iran; ^3^Social Determinants of Health Research Center, Mashhad University of Medical Sciences, Mashhad, Iran; ^4^Department of Epidemiology and Biostatistics, School of Health, Mashhad University of Medical Sciences, Mashhad, Iran; ^5^Health Education and Health Promotion, Department of Health Education and Health Promotion, School of Health, Mashhad University of Medical Sciences, Mashhad, Iran

**Keywords:** child marriage, qualitative research, socio-ecological model, policy making, cultures, interpersonal relations

## Abstract

**Introduction:**

Girl child marriage is an example of a human rights violation, the unfortunate consequences of which have raised growing concerns in the health system and have become a development challenge. Therefore, this research was conducted to identify the factors driving girl child marriage based on the Socio-Ecological Model.

**Methods:**

The present qualitative study employed guided content analysis and a deductive approach to identify the driving factors behind girl child marriage in Bam City. Sampling was targeted among Women aged 15–30 got married under the age of 15, family members, informants, and policymakers. Data were collected through semi-structured interviews and analyzed using Hsieh and Shannon approach and MAXQDA software.

**Results:**

The data was classified into five categories: individual, interpersonal, organizational, community, and society. Individual factors were classified into biological, psychological, and demographic factors. Interpersonal factors were categorized into two categories: family structure, Ineffective interactions and social support. Organizational factors included Weakness in the education and care system. Community factors included the economic situation governing the society. The society factors were divided into two categories: Cultural and social factors governing the society, and Weakness in policy making and legislation.

**Discussion:**

The findings showed that the socio-ecological model is a suitable framework for explaining the driving factors of child marriage. Girl child marriage is not merely a personal or familial decision; rather, it is influenced by the interactions of different systems that can either exacerbate or mitigate the issue. Consequently, the management, control, and prevention of Girl child marriage necessitate comprehensive efforts at regional, national, and international levels. In addition to creating platforms for the empowerment of girls at both national and regional levels, international policies can also foster a supportive environment.

## 1 Introduction

Girl Child Marriage (GCM) is a global social phenomenon in which teenagers under the age of 18 get married (1). This practice is increasingly recognized as a significant barrier to global health, development, and gender equality (2). GCM is linked to a wide array of physical, social, and economic consequences, jeopardizing the lives and health of girls. The lack of physical, physiological, and psychological readiness for marriage and motherhood places girls at heightened risk for cervical cancer (3, 4), sexually transmitted diseases (STDs) (5), malnutrition (6), and chronic illnesses in adulthood (7). These girls are also more likely to experience domestic violence (8). Evidence indicates that they face a greater likelihood of developing various mood, anxiety, and other psychiatric disorders in adulthood (9, 10). Furthermore, they are at an increased risk of depression, suicide, and divorce compared to those who marry later (11, 12). Limited educational opportunities and a lack of decision-making power restrict their access to contraceptive methods (13). Consequently, they often encounter unintended pregnancies (14), which elevate the risk of miscarriage and having multiple children (15, 16). They are also more susceptible to obstetric complications (17). Additionally, the marriage of girls has intergenerational repercussions for the well-being of their offspring. Early marriage among mothers is associated with preterm births, low birth weights, short stature, underweight conditions, and higher infant mortality rates (18, 19) If left unaddressed, GCM imposes significant economic costs on society, amounting to billions of dollars (20).

Globally, an estimated 12 million girls are married at an early age each year (21). It is projected that by 2030, more than 120 million girls will be married before the age of 18 (22). One in five girls will enter into marriage before reaching 18, either formally or in an informal union, with nearly 18% of them being under the age of 15 (23). GCM is not confined to any specific region or culture; however, it is most prevalent in South Asia and Africa, particularly in the poorest and most rural areas (24). Among women aged 20–24, 33% in West Africa, 26% in South Asia, 21% in America and the Caribbean, 16% in the Eastern Mediterranean, 8% in Eastern Europe and Central Asia, and 19% in the world were married for the first time before the age of 18 (25).

Of the 650 million child brides globally, 47 million reside in the MENA (Middle East and North Africa) region, accounting for 6% of the total. Each year, 700,000 girls in the MENA region are forced into marriage before the age of 18. Sudan (34%), Yemen (32%), and Iraq (24%) have the highest rates of GCM in the region, while Iran and Egypt rank fourth with 17% (26). Unfortunately, there are no reliable and official statistics on the prevalence of GCM in Iran. In a study, Azimi reported that the GCM rate in Iran was 12.35 percent in 2011 and decreased to 11.21 percent in 2016 (27). Despite the increasing global consensus that GCM must be prevented due to its detrimental effects on girls’ rights and well-being, no region is currently on track to meet the Sustainable Development Goal (SDG))Target 5.3(of eliminating all harmful practices, including child, early, and forced marriage (2), and there are research gaps in the trends, prevalence, and determinants, and correlates of child marriage (28).

### 1.1 The socio-ecological model

GCM is a phenomenon influenced by various factors that cannot be fully understood from a single perspective (29, 30). The Socio-Ecological Model (SEM) is a multi-level approach used to comprehend systemic effects in health-related issues and pinpoint intervention opportunities. This conceptual model is widely embraced by health promotion researchers. The Ottawa Charter also underscores the importance of integrated actions at the individual, community, and societal levels to enhance health promotion (31). Individual level are micro-level factors that include personal history issues that predispose a girl to early marriage. Such as Lack of information regarding health and reproductive issues. The interpersonal level are factors that increase risk of a girl getting married early as a result of how she relates with peers, family members, and teachers. The community level are factors that increase risk based on community and social environments, especially schools and neighborhoods. Society level are larger, macro-level factors that influence GCM and include, social policies that create or sustain gaps between groups of people (32).

Who ultimately makes the final decision regarding a girl’s marriage? This decision-making process is significantly influenced by cultural beliefs. In patriarchal societies, the decision regarding marriage is typically made by the head of the family, often a male figure such as father and the paternal grandfather (33). Forced marriage exemplifies this type of decision-making, as highlighted in various studies (34, 35). However, in some societies, girls play a role in determining their own marriage choices.

In Iran and around the world, numerous studies have been conducted on the subject of GCM. In Mirzaei’s et al. study, stakeholders identified social stigma prevention, religious beliefs, virginity preservation, and legislation as the primary factors driving GCM in North Khorasan, Iran (36). Bozorgi’s et al. research revealed that tradition, poverty, satisfaction of sexual needs, and escaping from the home environment were the main drivers of GCM in Tehran (37). Çelik’s et al. study emphasized family coercion, love, poverty, and the losses experienced during the war as the most significant factors leading to early marriage among Syrian immigrant women (38). In her study, Kohno et al. employed a SEM to identify the causes of GCM in Kelantan, Malaysia. She identified immaturity in decision-making, family poverty, and religious and cultural norms as the primary factors contributing to GCM (39). Psaki et al. identified several factors contributing to child marriage in Bangladesh, Malawi, and Niger, including social norms, poverty, fear of sex and pregnancy among girls, lack of opportunities, and lack of agency (40).

In our initial review, we found that few studies have employed a conceptual framework to examine the factors influencing child marriage, and none have illustrated the relationships and interactions among these factors. Most studies have concentrated on the marriage of girls under 18 years of age. To date, no research has been conducted in Bam, a unique location characterized by a high frequency of child marriage among those under 15 years of age, as well as cultural, ethnic, and religious diversity. Additionally, its proximity to Hormozgan and Sistan and Baluchestan provinces, where polygamy and early marriage are prevalent, further distinguishes Bam.

Therefore, this study contributes to our understanding in several ways. First, it offers a structured conceptual framework for the factors influencing child marriage. Second, it identifies the drivers behind the marriage of very young women under the age of 15 in the Iranian context, which may also be applicable to other regions of the world with similar socio-cultural gender norms.

## 2 Methods

### 2.1 Study design

In this study, we employed a naturalistic paradigm and a qualitative approach, both of which are common methods in humanities research. In this methodology, facts are uncovered by examining culture, foundational values, beliefs, and other related aspects. Employing a quantitative approach that adheres to the positivist paradigm—asserting that individuals can discern reality through their senses and objective descriptions of variables—was inconsistent with the study’s objectives. Consequently, we utilized a qualitative approach to identify the factors influencing GCM based on the SEM.

In this study, the ontological position of critical realism was adopted. According to this perspective, reality can be comprehended by analyzing the human mind and socially constructed meanings (41). We used this approach to determine the drivers of GCM based on the SEM and followed the guidelines of Standards for Reporting Qualitative Research (SRQR) (42) (Supplementary Table S1). This study aimed to address the following question.

- What are the individual factors leading to GCM?
- What are the interpersonal factors leading to GCM?
- What are the organizational factors leading to GCM?
- What are the community factors leading to GCM?
- What are the society factors leading to GCM?

### 2.2 Study methods

In this study, we employed in-depth interview (IDI) and key informant interview (KII) as our primary data collection methods. These approaches are among the most widely used in qualitative research (43). Our decision to select these methods was driven by their ability to meet our research objectives. The advantages of in-depth interviews include flexibility, the freedom granted to the interviewer, the ability to thoroughly explore topics, the opportunity to gain a comprehensive understanding of participants’ responses, and the provision of explanations for terms and phrases (44) that help achieve the interview’s objectives.

### 2.3 Study setting

Kerman province in Iran is one of the least developed provinces, ranking 15th among 31 provinces in terms of the Human Development Index (HDI) (45). Bam city, covering an area of 18,000 square kilometers (46) and with a population of 228,241,000 people (47), is one of the southern cities of Kerman province where GCM is prevalent (48). This city has a hot and dry climate and is bordered by the provinces of Sistan and Baluchistan, Hormozgan, and South Khorasan. Its economy relies on agriculture, date production, and automobile and component factories. The majority of the population is of Fars ethnicity with a Baloch minority, and in terms of religion, most are Shiites with a Sunni minority. In 2003, a devastating earthquake claimed tens of thousands of lives, resulting in significant changes in the economic, social, and structural landscape of the region.

### 2.4 Participants and recruitment

This study utilized a qualitative approach of content analysis to elucidate the driving factors of GCM based on the SEM. Sampling was purposefully conducted among women who married under the age of 15, family members, informants, local policymakers, and key policymakers from April 2023 to January 2024. The study focused on women who had experienced marriage before the age of 15, family members who were the primary decision-makers in GCM (mothers, fathers, husbands), key policymakers (Members of Parliament) responsible for legislating the marriage age of girls and boys, and local policymakers involved in relevant decisions. In line with the SEM, teachers who have direct contact with students, healthcare providers involved in adolescent health programs, and other informants such as sociologists and lawyers were considered part of the main target group.

### 2.5 Inclusion and exclusion criteria

The inclusion criteria for the main target group, child brides, included women aged 15 to 30 who were married before the age of 15. To eliminate the social effects of the earthquake on child marriage, the time of marriage was considered 5 years after the earthquake and to obtain maximum diversity in the sample, a time span of 15 years was considered (2008–2023). Being Iranian, native to the region, and providing consent to participate in the study were other inclusion criteria for the girls (49, 50), considering that marriage under the age of 15 is allowed in Iranian law (51), but it is not supported by custom and society, we made marriage under the age of 15 a criterion. The inclusion criteria of policymakers and informants included being involved in GCM, having at least 5 years of work experience, and agreeing to participate in the study. The inclusion criteria of family members included consenting to participate in the study. In each group, individuals who did not consent were excluded from the study (Supplementary Table S2).

### 2.6 Data collection tools and procedures

We collected data from participants through semi-structured interviews and face-to-face methods. The interviews were conducted by a local person who was familiar with the cultural and social conditions of the region (first author). Five versions of interview guides were developed by reviewing the research literature on GCM for women, family member (parents, husband), policymakers, informant (teacher, healthcare provider, other). The questions were designed based on the SEM, encompassing individual, interpersonal, organizational, community, and society sectors. After the pilot tests, minor changes were made to the interview guide and some questions were rewritten based on the participants’ feedback to make it easier for them to answer. For example, eligible girls were asked how they decided to get married at a young age. Family members were asked, how did you decide that your daughter should get married at a young age? The informants were asked what is your opinion about GCM. The policy makers were asked how do you evaluate the laws and policies of the society regarding GCM? The interview guide for the five groups is presented in Supplementary Table S3.

The interview was conducted with the coordination of the participant at school, health center, workplace. Before the interview, the objectives of the research were explained, and consent was obtained for audio recording. They were assured that the information would remain confidential with the researcher, and the audio file would be deleted after the results were extracted. Informed consent forms were obtained from all participants. Because the girls were under 15 years old, Iranian law designates husbands as the legal guardians of their wives. Consequently, two consent forms were collected from eligible girl: one written consent from the girl themselves and another from their husbands. After conducting 48 interviews, no new concepts were discovered. The codes appeared to be repetitive and saturated. To ensure data saturation, we conducted three additional interviews, bringing the total number of interviews to 51. The interviews were conducted between 45 and 60 min.

### 2.7 Reliability

The following strategies were employed to enhance validity, confirmability, dependability, and reliability based on Lincoln and Guba’s criteria (52). The researcher’s tasks included eliminating personal opinions, ensuring diversity in participant selection, dedicating sufficient time for interviews, continuing interviews until data saturation was achieved, documenting interviews in detail right after, immersing deeply in the data, accurately extracting codes, and providing a thorough, step-by-step account of the research process along with seeking guidance from professors.

### 2.8 Data analysis

Data were analyzed in three stages: preparation, organization, and reporting, following the methodologies of Hsieh and Shannon (53) and Elo (54). In the preparation stage, interviews were transcribed, and each transcript was read multiple times to gain a deeper understanding. Next, in the organization stage, the researcher created a matrix to identify the main concepts. To locate content that corresponded to the previously defined categories, the data were reviewed several times, and initial codes were established based on the findings. Additionally, other meaningful units that were not directly related to the defined main categories but were generally associated with the phenomenon under study were integrated into the existing main categories of the matrix or used to form new main categories based on their conceptual and logical relevance. In the reporting stage, the data were presented according to the SEM, organized into categories, subcategories, and open codes. An independent researcher was involved to ensure the accuracy of the review and the process of generating codes and categories. In cases of disagreement between the primary researcher and the independent researcher, a third researcher was consulted to make the final decision. Microsoft Word software was utilized for interview transcription, and MAXQDA software was employed for data analysis.

### 2.9 Measuring demographic variables

Age and work experience were measured in years. Education levels were classified into five categories: illiterate, under diploma, associate diploma, bachelor’s degree, and master’s degree or high degree. Participants’ Job were categorized as Member of Parliament, City and Provincial Management, Missionaries of Religion, teacher/ Consultant, Health Care providers, Homemaker, and other. Participants were divided into two groups based on ethnicity: Fars and Baloch; two groups based on religion: Shia and Sunni; and two groups based on location: urban and rural. Participants assessed their economic status as good, poor, or Medium (Table 1).

**Table 1:** Demographic characteristics of study participants.

Variables	Girls	Family member	Informant	Local and key policy makers
	Frequency (%)	Frequency (%)	Frequency (%)	
Age (year), Mean (SD)	21.80 (4.76)	43.62(13.45)	42.56(5.57)	50.57(3.86)
Marriage age (year), Mean (SD)	13.80 (0.4)	–	–	–
Duration of marriage(year), Mean (SD)	8 (4.7)			
Work Experience (year), Mean (SD)			18.18(5.15)	23.71(4.78)
Gender				
Male	0	7(53.84)	3(18.8)	6(85.7)
Female	15(100)	6(46.15)	13(81.3)	1(14.3)
Education level				
Illiterate	0	1(7.69)	0	
Under Diploma	8(53.3)	3(23.07)	0	
Diploma	6(40)	5(38.46)	0	
Associate or Bachelor’s Degree	1(6.6)	3(23.07)	8(50)	
Master’s Degree or High degree	0	1(7.69)	8(50)	7(100)
Job				
Member of Parliament				2(28.6)
City and Provincial Management				5(71.4)
Missionaries of Religion			2(12.5)	
Teacher/ Consultant		1(7.69)	8(50.1)	
Health Care providers			3(18.8)	
Homemaker	13(86.6)	4 (30.76)	0	
Other	2(13.3)	8 (61.53)	3(18.75)	
Ethnicity				
Persian (Fars)	12(80)	11(84.61)	15(93.75)	
Baloch	3(20)	2(15.38)	1(6.25)	
Religion				
Shia	12(80)	11(84.61)	15(93.75)	7(100)
Sunni	3(20)	2(15.38)	1(6.25)	
Residence				
Urban	8(53.3)	6(46.15)	15(93.75)	7(100)
Rural	7(46.6)	7(53.84)	1(6.25)	
Economic Situation				
Good	5(33.3)	8(61.53)	16(100)	7(100)
Medium	7(46.6)	3(23.07)	0	
Poor	3(20)	2(15.38)	0	
Total	15(100)	13(100)	16(100)	7(100)

## 3 Results

A total of 51 eligible people participated in this study. The participants included 15 (29.41%) girls, 13 (25.49%) family members, 16 (31.37%) informants, and 7 (31.72%) policymakers (Table 1). A total of 1,275 codes were extracted. After merging similar codes, 213 codes, 22 subclasses, 7 classes, and 5 levels (individual, interpersonal, organizational, community, and society) were obtained. At the individual level, biological, psychological, and demographic factors were extracted. At the interpersonal level, two levels of family structure, dysfunctional interactions, and social support were identified. The organizational level was categorized as weak in the educational and care system. At the community level, the economic status of the ruling class was examined. The society level included socio-cultural factors governing the society and weaknesses in policy-making and legislation. The following is a summary of the results obtained in each section. More details are shown in the Table 2.

**Table 2:** Categories, subcategories and codes obtained from the interviews.

Category	Subcategory	Code
1.Individual factors		
1.1. Biological, psychological and demographic factors	1.1.1. Insufficient cognitive and inferential development	Instant decision-making, aimlessness in life, childish thoughts of love and infatuation, responding to the need, misunderstanding of common life, ineffectiveness of education, disappointment in education, lack of knowledge about marriage and pregnancy, lack of knowledge about individual rights, weakness of life skills.
	1.1.2. Physiological and anatomical features	Tall, large body, menstruating, beauty, sex attractiveness, female anatomy, early puberty
	1.1.3. Facing stressful factors in life	Tension in the family, difficult living conditions, insecurity in the family, limited independence and freedom, experiencing failure, feeling lonely
	1.1.4. Demographic characteristics	Baloch ethnicity, Sunni religion, living in the rural, living in the outskirts of the city, nomadic life, living in tropical areas, family size
2. Interpersonal factors		
2.1. Family structure	2.1.1. Traditional parenting methods	Restrictions on girls’ social relationships, strengthening traditional roles, making girls responsible for growing up, encouraging and supporting the family, making decisions by parents, accepting suitors at a young age, accepting part of the responsibility of couples.
	2.1.2. Family values	Having a social, economic and cultural status, similar social status and being of the same class, having sufficient knowledge of girls and boys, being chaste, being a religious girl, being a responsible, housewife, being committed, high social relations, with modesty.
	2.1.3. Family breakup	Unrestrained family members, divorce, imprisonment, violence, addiction, adoption, misbehaving with a girl, arguments in the family, death of parents, physical and mental disability in the family.
	2.1.4. Inefficiency of management and problem solving in the family	Escaping from responsibility, not paying attention to education, providing financial resources by the mother, power and influence of the mother, disagreement between parents, the desire to expand the family, fear and worry of the family, loneliness of the mother, prevention of family disputes, acceptance of the son-in-law as a son.
	2.1.5. Weak social capital in the family	Weak social relations of the family, low education of parents, low attachment among family members, ignorance of parents, absence of one parent at home, false job, parents with only one child, only one daughter, not caring about the child, lack of trust in the family.
2.2. Ineffective interactions and social support	2.2.1. Peer pressure and reference groups	Stimulation of emotions, recommendation of peers, competition with peers, support of religious leaders, approval of relatives and relatives, media advertising.
	2.2.2. Inappropriate caring and supportive teacher-student relationships	Counselor inefficiency, lack of motivation of the teacher, embarrassment of the student, weakness in responsibility, collective counseling
3. Organizational factors		
3.1. Weakness in the education and care system	3.1.1. Lack of training and empowerment programs	Lack of educational programs in schools, lack of educational programs in health centers, lack of curriculum, lack of education in the family, lack of education through organizations, lack of distribution of books and manuals.
	3.1.2. Lack of social services	Lack of lawyers supporting children, lack of children’s rights protection organization, weak support of researchers for women’s rights
	3.1.3. Weakness in organizational and inter-organizational coordination	Inappropriate organization of marriage counseling (instead of pre-marriage counseling, it is during marriage), lack of coordination to implement control strategies, lack of comprehensive training in beneficiary organizations, lack of joint research in the field of child marriage, lack of coordination to determine growth certificate, lack of cooperation for documentation, top-down management, punishment-based management, lack of data source, ambiguity in “child definition” in organizations, boycott of facts, non-expert force, lack of sufficient time for)judgment, school counseling (, lack of inter-departmental cooperation in issuing court verdict, lack of expert force (psychologist), statism, quantitative evaluation of performance judges, lack of human resources, insufficient budgets, carelessness in issuing verdicts, inadequate supervision
4. Community factors		
4.1. The economic situation governing the society	4.1.1. Financial Problems	Reduction of financial burden, financial benefits, Inability to cover living expenses, inability to pay for education, more welfare, financial support of spouse.
	4.1.2. Economic instability	Inflation, economic instability, economic sanctions.
5. Society factors		
5.1. Cultural and social factors governing the society	5.1.1. Common religious and cultural attitudes and beliefs	The sin of premarital sex, belief in fate, getting married is easy, marriage at the age of duty and maturity, following the tradition of the prophet, religious prejudices, perfecting the religion of magic, chances, to shape the behavior of the daughter, obedience to the husband, attracting girls to the workforce, increasing the social power of the family, increasing the population family, more influence in the group and region, polygamy, culture of collectivism, imitation of saudi sunni scholars, more compatibility, prestige, achieving more success, successful pregnancy, gender discrimination, patriarchy, social pressure.
	5.1.2. Adherence to old traditions	Family marriage, respecting relatives, following customs (sweets eaten), being similar to others, preventing damage to the daughter, preserving honor, preventing social stigma, vying, social acceptance, too much suitor insistence, gift to the mother, tribal decision-making, the unusualness of rejecting a suitor, transplanting for blood price, preserving honor. Unrestricted relations, customary norms, involvement of relatives.
	5.1.3. Vulnerability of society	Natural disasters occurred (earthquake), drug transit route, crime-ridden area (Crimes related to drug distribution), proximity to Afghanistan and Pakistan, cultural influence from Afghanistan and Pakistan, underdevelopment, social damage (drug addiction, divorce), migration, insecurity, drought.
	5.1.4. Social changes	The spread of new entertainment, the expansion of social networks, access to the Internet, access to Android phones, bombardment of information.
5.2. Weakness in policy making and legislation	5.2.1. Inefficient social policies	Deficiency in marriage and childbearing policies (population youth policy) granting facilities, banning the education of sexual issues in schools, guidelines for the simultaneous education of married and unmarried girls in schools, a solution to deal with the increasing age of marriage, a solution to deal with the aging of the population.
	5.2.2. Absence of restrictive laws	Absence of an explicit law on the age of marriage, legal loopholes, customary laws, no prohibition of marriage under 15 years, no fines and punishments, ease of approval of growth orders in legal authorities, prohibition of premarital sex, bypassing laws and regulations, lack of comprehensiveness of laws, negligence in the implementation of one-dimensional laws The presence of inconsistency laws in civil laws, laws based on Shariah, experimental laws, inappropriate criteria for marriage approval, lack of standards for issuing judgments, the growth of one-dimensional concepts in civil laws, expediency in law enforcement.

### 3.1 Driving factors of GCM at the individual level

#### 3.1.1 Biological, psychological and demographic factors

##### 3.1.1.1 Insufficient cognitive and inferential development

This category refers to the characteristics of girls’ cognitive system that make them prone to marrying at a young age. Despite lacking knowledge about marriage, pregnancy, individual rights, and life skills, most of the girls quickly decide on marriage or leave the decision to their parents. In most cases, girls did not want to continue their education; their most important goal in life was to start a family. Discussing weddings, intimacy, and fulfilling the needs of girls was a dream that came true with marriage.

*A girl said: “At that time, I wanted to get married, wear a wedding dress, and dye my hair” (29 years old, age at marriage 14 years,city)*.

##### 3.1.1.2 Physiological and anatomical features

In some societies, in accordance with social norms, having a tall body, a large body, a female anatomy, and menstruation are considered as signs of girls’ readiness to marry and start a family. In addition, beauty and sexual attractiveness were also driving factors for GCM.

*A family member said: “Although my daughter was 14 years old, she was tall and had a big body like this” (father, 51 years old, freelancer)*.

##### 3.1.1.3 Facing stressful factors in life

Freedom from challenging living conditions was also a contributing factor to early marriage among girls. These conditions may arise from a lack of independence and freedom. Girls perceive decisions regarding social interactions and clothing choices as examples of independence and freedom. In societal traditions and customs, clothing plays a significant role, and parents often strive to uphold these traditions by regulating the attire of girls. If they view their family as a hindrance to attaining these freedoms, they may be motivated to marry in order to break free from their current situation and pursue their aspirations. Another factor that led to a difficult life was being the only child of a single mother, which made girls feel lonely.

*An informant said: “some families prevent girls’ relationships; they do not allow them to go out with friends. They control how they dress. They do not let girls dress as they please (female, consultants, 25 years of work experience)*.

##### 3.1.1.4 Demographic characteristics

Ethnicity and religion are important demographic variables. The city of Bam is home to Persian and Baloch people in terms of ethnicity, and Shia and Sunni religions in terms of religion. In the current region, it appears that the prevalence of girls getting married under the age of 15 is higher among the Baloch and Sunni communities. It is worth noting that within the Islamic faith (Shia and Sunni), there are recommendations supporting early marriage for girls, which unfortunately can result in GCM s. Girls who marry before the age of 15 often find it difficult to continue their education due to pregnancy, and in most cases, their education is limited to high school. Another demographic factor is the place of residence. People who live in less privileged areas such as rural areas, outskirts of the city, or are nomadic are more likely to marry at a young age for several reasons. The typical occupations in rural and nomadic areas are animal husbandry and agriculture, and marrying girls at a young age is crucial for providing labor. Adherence to old traditions is more common in rural areas. Going against the traditions and customs of the society can lead to criticism from the community. Villagers typically have more children and tend to marry at a younger age. Financial incentives and the desire to alleviate financial burdens are additional factors that encourage early marriage in rural communities. In the outskirts of the city, basic life facilities are often lacking. Social norms differ from those in other areas. In some cases, many relationships in these areas expose girls to early marriage. Usually, a segment of the marginalized population consists of criminals and addicts. In these neighborhoods, residents frequently interact with one another to exchange drugs. Additionally, girls from these families often attract attention and are subjected to selection, with their families not objecting to their marriages in order to maintain these relationships.

*An informant said:” The areas that are close to Sistan and Baluchistan, their culture leads to marriage at a young age, most of them are Baloch and Sunni (female, lawyer, 19 years of experience work)*.

### 3.2 Driving factors of GCM at the interpersonal level

#### 3.2.1 Family structure

##### 3.2.1.1 Traditional parenting methods

In rural areas, parents often employ the parenting techniques passed down from their own parents. Girls are introduced to potential suitors at a young age and are taught essential household tasks like cooking and housekeeping. Parents hold the belief that girls should experience life’s challenges and take on responsibilities similar to those of their mothers. In this traditional educational approach, the decision to marry is typically made by parents and respected family elders.

*A local policymaker said:” Some families see marriage as a way to make their daughters responsible and teach them how to live in a home” (male, Provincial Government, 17 years of experience work)*.

##### 3.2.1.2 Family values

The family of a boy and a girl viewed family values from two different perspectives. The girl’s family emphasized factors such as social, economic, and cultural status, similarity in social standing, and the suitor ‘s level of education. They believed that having a “good suitor, a favorable social, economic, and cultural standing, justified marrying off children at a young age.

*A girl said: “My suitor was rich and had morals. He was the one I loved. There was no reason for me not to marry” (20 years old, age at marriage 13 years, city)*.

On the other hand, one of the most important reasons for husbands to marry young girls is to get to know the girl well enough, ensuring that she is chaste, religious, a housewife, responsible, committed, and has high social relations. The integration of girls into society is linked to their knowledge and learning, which may make them less outspoken. Therefore, marrying young girls who have not yet entered society and are certain of their purity and decency is considered a suitable option.

##### 3.2.1.3 Family breakup

In cases such as divorce, imprisonment of parents, death of parents, and violence in the family, the family foundation is weakened, and girls do not receive the support of their parents, providing the groundwork for their marriage. It seems that the earthquake of 2003, which was accompanied by the abandonment of a large number of children, has continued to impact children’s marriages even after a decade.

*A girl said: “I got married because I was motherless. My mother died in an earthquake, my father was an addict, he raised us all, I was still a child, I did not want to, but I had no choice” (27 years old,* a*ge at marriage 14 years, city)*.

Additionally, the physical and mental inability of parents to care for their daughters forces them to agree to their daughters’ marriages.

*A mothers said: “His father had a heart operation; he was worried about him. He said that if I die, they will say that he is fatherless and we should marry him” (code 19, 42 years old, housewife)*.

##### 3.2.1.4 Inefficiency of management and problem solving in the family

Various examples of dysfunctional management and problem-solving in the family were expressed by the participants, which can be the basis for the marriage of girls under the age of 15. For example, GCM was considered a way to alleviate parents’ worries, protect girls from harm, and provide care for them. Sometimes, girls had multiple suitors, and parents agreed to marry them off at a young age to end these visits and avoid family disputes. Another decision by families was the longing for grandchildren and the desire to overcome loneliness. Fathers and mothers who married late in life or lost family members in an earthquake showed a greater inclination to marry off their daughters at a young age.

*A family member said: “We just had a daughter; we married her early so that we could have grandchildren” (father, 57 years old, military force)*.

##### 3.2.1.5 Weak social capital in the family

Factors such as the number of children, weak social relationships, false parental employment, the absence of one parent at home, and low attachment among family members threaten the structural social capital of the family. Parental ignorance and low educational parent, along with a lack of care from the mother, undermine the cognitive social capital of the family and erode trust within the household. This undermines the protective role of the family and increases the vulnerability of its members, thereby providing a foundation for child marriage. In families with few children, relationships among family members are limited, resulting in a small family support network that can negatively impact social capital. Conversely, in families with many children, although relationships among family members may be more extensive, there is a higher likelihood of child neglect, which can diminish the attachment between family members.

*A local policymaker said: “Families that marry their daughters off early usually have weak relationships. There is no warm relationship between them” (male, City council member, 20 years of work experience)*.

#### 3.2.2 Ineffective interactions and social support

##### 3.2.2.1 Peer pressure and reference groups

The most crucial interactions for teenagers occur within the family, school, and society. By introducing guidelines for the simultaneous education of both married and unmarried students, they will have more exposure to married life experiences, potentially influencing them to marry at a young age. In traditional societies, there is a societal expectation for girls to marry. Religious leaders and media campaigns also promote early marriage for girls. In Islam, marriage is permissible after puberty, and it is encouraged to eliminate obstacles that may hinder marriage, such as imposing a heavy dowry and minimizing extravagant wedding expenses. The promotion of simplified marriage by religious leaders was one of the reasons cited by participants.

*A family member said: “When we go to the mosque, they keep telling us to take it easy on marriage, it will be difficult for a girl to raise children when she gets older” (mothers, 44 years old, housewife)*.

##### 3.2.2.2 Inappropriate caring and supportive teacher-student relationships

The results showed that the lack of appropriate care and support relationships between teachers and students provides the basis for girls getting married under the age of 15. Part of the weakness in the teacher-student support system is linked to the performance of both teachers and counselors. The shortage of human resources restricts the availability of counselors in schools, as they are only present for a few hours each week. Additionally, the quantitative evaluation system has compelled counselors to focus on documenting their performance during these limited hours to achieve an acceptable score. They must minimize the time spent on documenting and instead concentrate on counseling and guiding students. Another aspect of this issue pertains to the students themselves. Some students feel embarrassed discussing marriage with a counselor, while others refuse to seek counseling due to previous negative experiences.

*An informant said: “School counselors should minimize the time spent on paperwork, their main task is to counsel the children, while he comes two days a week, they are only busy with paperwork. They should seek documentation They have to fill out many forms and collect documents to submit reports to higher levels, which takes up a lot of their time” (female, school deputy, 18 years of experience work)*.

### 3.3 Driving factors of GCM at the organization level

#### 3.3.1 Weakness in the education and care system

##### 3.3.1.1 Lack of training and empowerment programs

Even though education is the main pillar of empowering girls, the results showed that targeted education of girls is neglected by organizations. In schools, the usual education is ongoing, but effective formal and informal education about marriage is not provided. Health care centers do not prioritize the education of teenage girls who are likely to get married. Their education in schools is sporadic and only occurs when educational instructions are issued by the health deputy. Families also fail to educate girls about marriage, childbearing, and related issues. Consequently, girls enter married life without receiving adequate educational content and information about marriage and childbearing.

*An informant said: “We do not talk about marriage. We do not have a special plan for teenagers. When there is an occasion, for example, we go to school for mental health week, we talk to them” (female, health care worker, 14 years of experience)*.

##### 3.3.1.2 Lack of social services

The participants stated that there are no governmental or non-governmental social service providers capable of supporting and preventing GCM. This is mainly due to laws permitting marriage under the age of 15. Consequently, many initiatives to support girls are either not initiated or halted. Lawyers are prohibited from advocating for girls’ rights. Researchers lack sufficient support to investigate the issue of GCM and its profound personal and social repercussions. Non-governmental organizations are not established in this area.

*An informant said:” if there are many people like you who are doing research, there will be people who defend women’s rights, the situation will be different (female, teacher, 28 years of experience work)*.

##### 3.3.1.3 Weakness in organizational and inter-organizational coordination

The findings revealed that the prevention and control of GCM under the age of 15 is not a priority for any of the relevant organizations (University of Medical Sciences, Education Organization, Judicial System, etc.). Even within the structure of services provided by these organizations, there are notable shortcomings. For instance, couples often seek marriage counseling only shortly before their marriage is officially registered. This delayed counseling regarding the marriage process, pregnancy, and its implications prevents girls from having the opportunity to reconsider their decisions. Furthermore, even this late intervention is often insufficient to dissuade couples with genetic concerns from proceeding with marriage.

In other related organizations, such as the judicial system, factors including a lack of human resources, performance evaluations based on quantity, insufficient budgets, and inadequate supervision, carelessness in issuing verdicts also impact the marriage of young girls. Furthermore, lack of coordination to implement control strategies, joint training, joint research, issuing restraining orders, and documentation are additional factors influencing GCM. The top-down management style in organizations also hinders employee performance and exposes them to organizational penalties.

*A local policymaker said: “How many places can we connect with the people? How many places can we connect with people? Schools, medical universities, health centers should work together to educate children, but there is no cooperation” (male, Deputy Governor, 20 years of experience work)*.

The absence of a common definition of “child” poses another challenge for organizations and can heighten the vulnerability of girls. For instance, clerics argue that 15-year-old girls who reach adulthood are no longer considered children. Teachers were reluctant to refer to 15-year-old girls as children.

*An informants said: “I do not think child marriage is the right word. When a girl reaches puberty and starts menstruating, it shows that she is not a child. 15-year-old girls should not be called children” (male, clergyman, 16 years of experience work)*.

### 3.4 Driving factors of GCM at the community level

#### 3.4.1 The economic situation governing the society

##### 3.4.1.1 Financial problems

Families who were unable to meet the cost of living welcomed suitable suitors. They arranged marriages at a young age to achieve more prosperity. Some believed that rejecting a suitor would jeopardize their chances of having a good life. Not having the burden of education costs compelled the girls to opt for marriage, aiming to alleviate the family’s financial strain and embark on a new chapter in the girls’ lives.

*A girl said: “We live in the village; my father did not have money to buy services for us to go to school. When we stayed at home, we should have gotten married” (age at marriage 14 years, 24 years old, village)*.

##### 3.4.1.2 Economic instability

policymakers believe that economic conditions have a significant impact on social events and decisions. The absence of proper economic structures, which results in inflation and economic instability, affects the ability to cover family expenses and may lead families to marry off their daughters at a young age. Economic sanctions imposed on Iran have posed a significant challenge to the economic situation of the society. These sanctions have led to heightened inflation and economic instability, severely restricting families’ spending capabilities.

*A local policy makers said: “Now people are not in a good economic situation, the sanctions have an impact on people’s lives, prices change every day, families cannot meet the expenses, they are forced to marry their daughters at the age of 15” (female, lawyer, 19 years of experience work)*.

### 3.5 Driving factors of GCM at the society level

#### 3.5.1 Cultural and social factors governing the society

##### 3.5.1.1 Common religious and cultural attitudes and beliefs

In any society, behavior is influenced by cultural and religious beliefs. Religious and cultural attitudes and beliefs can be interpreted and analyzed from several aspects. Girls and families believe that marriage is fate, chances, or magic (Belief in prayers that force girls and their families to accept the suitor and make it impossible for them to express their opposition).

*A girls said: “I did not want to marry him at all. I kept crying, but I could not say no. They had prayed for me. I was speechless.”*

According to religious teachings, to fulfill one’s religious duties, one should marry at an appropriate age without strictness. In societies where tradition governs, individuals are pressured to adhere to social norms. Moreover, in patriarchal societies, men may resort to marrying children to uphold their dominance and control over women’s behavior. Patriarchal attitudes such as seeking compatibility for successful pregnancies are also prevalent in society.

*A family mamber said: “I believe that the girl can grow up with my own morals because she is young, I will teach her how she should be (husband, 27 years old, freelancer)*.

In rural communities and among the Baloch people, collectivism is the prevailing culture. In these societies, family size is considered a measure of social power and prestige. Polygamy is accepted according to religious and cultural standards, viewed as a means to consolidate family power.

*A local policy makers said: “In the Baloch people, it is believed that families with more children have more social power and will have more influence in that group and region” (male, education employee, 12 years of experience work)*.

##### 3.5.1.2 Adherence to old traditions

Although a set of traditions provides the grounds for GCM, it seems that the virginity of girls and preserving honor are among the most important factors. Families fear that girls may engage in premarital sex, leading them to consent to marrying off girls at a young age or arranging their marriage with a family member.

*A girl said: “When they see that the girl is being drawn to a bad path, it is better for the girl to get married in order to preserve the family’s reputation (29 years old*, *age at marriage 14 year, city)*.

Traditions such as bride price (a sum of money or quantity of goods given to a bride’s family by that of the groom, especially in tribal societies), making tribal decisions, marrying within the bloodline, and upholding honor, which were once prevalent in rural areas, have faded and are seldom observed today, though some participants still acknowledge them. In certain tribes, it is customary to accept the first suitor, particularly if they are introduced by the elders, rather than to reject them. In general, in any society where the marriage of girls is accepted at a young age, girls have suitors from childhood. Families, in order to conform to societal norms, avoid social stigma, and sometimes to compete with others, inevitably consent to the marriage of girls at a young age.

##### 3.5.1.3 Vulnerability of society

Natural disasters such as earthquakes, drug transit routes, crime-ridden area (Crimes related to drug distribution), proximity to Afghanistan and Pakistan, cultural influence from Afghanistan and Pakistan, social issues like drug addiction and divorce, migration, insecurity, and drought make society vulnerable and expose girls to early marriage. Recent droughts have prompted a significant migration from rural to urban areas. The high cost of living in cities influences families’ decisions regarding marriage for their daughters. Furthermore, early marriage is culturally accepted in rural communities, and the influx of migrants from these areas is perpetuating the practice of early marriage in urban settings.

The participants believe that proximity to Pakistan and Afghanistan causes cultural influence. In these countries, it is customary for girls to marry at a young age. This cultural practice has spread to the region. The proximity to the border also has other consequences. Drug trafficking contributes to a range of criminal activities and societal harms, including parental addiction, incarceration, and divorce. Children raised in such environments, particularly girls, are often at a higher risk of experiencing early marriage.

*A local Policymaker said:” Child marriage may be a cultural influence in the eastern provinces of the country, especially those bordering Afghanistan and Pakistan. Given the significant Afghan population in our country, some of their customs and traditions have an impact on us* (*male, City Council Member, 20 years of experience work*).

In addition, the earthquake that caused changes in economic and social structures, along with drought and migration from villages to cities, has had an impact on the prevalence of GCM.

##### 3.5.1.4 Social changes

The participants believe that social networks such as WhatsApp and Telegram provide a platform for making friends. Girls communicate with boys from a distance, which can lead to early marriage. The internet and social networks like Instagram expose girls to a wealth of information that they may not have the ability to evaluate. The unrealistic portrayal of life on Instagram immerses girls in a dream world and intensifies their desire for a different lifestyle.

*An informant said, “Students receive a significant amount of information from cyberspace that can influence their decision-making” (female, consultant with 18 years of experience work)*.

#### 3.5.2 Weakness in policy making and legislation

##### 3.5.2.1 Inefficient social policies

The participants mentioned some inappropriate social policies that facilitate the marriage of girls at an early age. Although the policies of keeping the population young do not directly address the marriage age of girls, they are effective in influencing the marriage of girls at a young age. Changing the reproductive age from 15 to 44 years to 10 to 55 years subtly communicates instructions to accept the marriage of girls under 10 years and provide promotional programs. Granting facilities to couples and providing an education platform for both married and unmarried girls in schools are among the policies that have been announced. These policies aim to encourage families and girls to delay marriage. On the other hand, the increase in the age of marriage and the aging of the population have led to young marriage being considered a solution to address the rising age of marriage and the challenges of old age. Typically, early marriage is linked to early fertility, which can result in a youthful population and serve as a protective factor against population aging.

*A local policymaker said, “The aging of the population is such a compelling factor that it encompasses all the* var*ious reasons for child marriage, so I approach this issue with tolerance” (male, Governorate, 17 years of experience work)*.

##### 3.5.2.2 Absence of restrictive laws

Legal loopholes regarding the age of marriage overshadow the deterrent role of the law. For instance, in civil law, there is no explicit mention of the minimum age for marriage. The current law neither prohibits marriage under the age of 15 nor imposes any fines or penalties for it. The ambiguity surrounding the age requirement for marriage creates legal loopholes that allow individuals to circumvent the law. Furthermore, the simplistic nature of the laws and concepts within the legal system challenges the principle of legal comprehensiveness, potentially enabling early marriages for girls. The participants stated that currently, the only criterion for confirming the marriage of girls at a young age is the growth certificate, which does not seem to be a suitable tool. In addition, there is no specific and appropriate standard for issuing growth certificates, and the judge makes a decision based on expediency and personal opinion about the girl’s marriage, which cannot be done well in most cases due to limited resources.

*An informant said: “I think the law should be amended. If the age of marriage is clearly specified in the law, no one can escape it. Of course, the legal age of marriage for girls should also be set above 15 years old” (female, lawyer, 19 years of experience work)*.

## 4 Discussion

GCM has become a significant public health challenge due to its numerous personal and social consequences. The international community is increasingly focused on controlling and preventing child marriage. Identifying the underlying factors of GCM within a conceptual framework can serve as a valuable guide for targeted and effective interventions. The study categorized the driving factors of GCM into five levels: individual, interpersonal, organizational, community, society.

The results indicate that inadequate cognitive and inferential development in adolescents, partly due to their biological and cognitive status and partly due to insufficient knowledge about marriage and fertility, can lead to child marriage. Piaget categorizes the stages of human cognitive development from childhood to adulthood into four distinct phases: sensorimotor, preoperational, concrete operational, and formal operational. He posits that even in the final stage, children are not yet capable of making decisions regarding significant life events, such as marriage (55). Research on teenagers’ perceptions of marriage supports this notion (56). Studies by Naghizadeh (57) and Eftikharzadeh (58) identify failure to reach intellectual and emotional maturity as a contributing factor to child marriage. At this stage, immaturity is masked by physical maturity, organ development, and menstruation, making girls appear ready for marriage (59–61).

This immaturity also impacts children’s decision-making abilities. Family restrictions that limit their freedom and independence, along with life’s challenges, compel them to escape their circumstances and marry. This finding has been corroborated by several studies in Iran (62–64), America (65), and Israel (66). Additionally, this study introduces restrictions on clothing and adornment as contributing factors. In other words, engaging in feminine activities such as applying make-up and wearing special dresses, which are typically forbidden for girls, is often referred to as “becoming a woman (67).

On the other hand, Baloch ethnicity and Islamic religion (Shia and Sunni) in terms of adherence to traditions and emphasis on girls’ virginity and chastity can be associated with early marriage. Other studies also support the effect of Arab (68), Ler (145), Kurdish (63), ethnicity on child marriage. Additionally, living in less developed areas, the outskirts of the city, and the countryside due to differences in socio-economic development and under the influence of factors such as education (69), access to the Internet, media, and information (70, 71), empowering girls and women (72), job opportunities (73), the reinforcement of traditional norms and beliefs (74), and vulnerability to natural disasters (49) provide grounds for early marriage.

In addition to individual characteristics, girls’ communication network, and most importantly family structure, had an effect on children’s marriage. According to the participants, most of the girls who got married under the age of 15 lived in a traditional family. According to McMaster’s model, the important dimensions of family members’ performance include problem-solving, communication, roles, emotional responsiveness, emotional intercourse, and behavior control (75). It is overshadowed by the traditionality of the family. Some experts believe that families with a traditional structure have problems in emotional issues, problem-solving, communication, assigning and responding to roles (76), and perform poorly (77). It seems to be part of the traditional view arising from the perceived place of women and girls in culture and society. Evidence shows that in the regions of Iran (63, 78), Bangladesh (79), Sudan (80), Uganda (60), social, economic, and livelihood issues are based on the traditional “mechanical society,” in such a way that male characters as the economic, livelihood, and security engine and women’s roles are limited to domestic work and childbirth. In this family, girls are prepared from childhood to play traditional roles such as cooking, taking care of children. Early marriage is an integral part of girls’ lives and is supported and encouraged. Families often strive to uphold these traditions and select a groom who matches their social standing. This choice can serve as an economic strategy, not solely based on personal preference, aimed at ensuring the welfare and happiness of the girls (81), or to gain advantages from child marriage (63, 70, 82, 83). For example, in Ethiopia, they exchange their daughters with an exchange called “macha” (that is, the man’s family pays money and cattle to the woman’s family) (33). Of course, it should be noted that these customs and viewpoints continue mostly in rural areas.

On the other hand, in traditional societies, parents are afraid that their daughter will get involved in an emotional or sexual love. Therefore, to find peace and freedom from worry, they agree to marry girls at a young age. They believe that after marriage, they are no longer responsible for their daughters. This finding has been confirmed in other studies as well (63, 79, 84). This perspective is not unique to Iran but is also prevalent in many Muslim countries such as Nepal (85), Bangladesh (86), and Syria (87). Family-arranged marriage is another tradition that encourages early marriage. Consanguineous marriage is reported not only in Iran but also in Egypt (88) Indonesia (89), Cameroon (90), Sudan (91), and Jordan (59).

In this study, examples of ineffective family management, such as addressing loneliness, the desire for grandchildren, and preventing family disputes that may lead to early marriage, were highlighted. This practice may be associated with parents’ low education and awareness (34, 92). Hasanah suggests that the low level of education impacts the comprehension of the essence and objectives of marriage, economic circumstances, environmental influences, personal choices, religious beliefs, the societal norms of early marriage, and adolescent engagement in free sex (93). Low education and occupation of parents also affect the social capital of the family. For instance, in rural areas, where parents are primarily engaged in agriculture and animal husbandry, they are often at home for fewer hours, which hinders family relationships. Envuladu (69) and Kalum (94) demonstrated that farmers typically have low education and socioeconomic status, which can lead to early marriage among girls. Moreover, factors like divorce, parental addiction, and imprisonment, which disrupt family cohesion, can also contribute to early marriage among children. This finding is supported by other studies as well (58, 63).

Despite the positive and negative impacts of the support network on the development (95) and health and well-being (96, 97) of children, they do not receive enough support either in the family or in society. Interaction with relatives by creating a competitive atmosphere a is not beneficial for them (67). In society, neighbors, elders, religious leaders, and the media (63), perpetuate the promotion of GCM (65, 98, 99). In school, peer support has a negative effect on marriage. Mengli (98), McDouga (61), and Tewahido (100) believe that excessive interest in friends and seeking their approval influences children’s decisions about marriage. Despite the importance of understanding students’ issues and problems and strengthening teachers’ support network (96, 97), the education system has not made significant progress in meeting students’ needs (101).

Participants have highlighted the inefficiency of support and care relationships in schools, such as the ineffectiveness of school counselors, lack of teacher motivation, and their weak sense of responsibility. Momeni also perceives the supportive role of the school as weak and believes that these relationships can lead to students losing interest in school and dropping out (102). In this context, the taboo surrounding sexual education poses a significant challenge to education in schools. Not only schools, but also families, organizations, and the media avoid addressing sex education, hindering efforts toward comprehensive education. In other instances, such as issuing certificates for child marriages, research development, providing control solutions, inter-departmental coordination are lacking. Evidence also shows that inter-sectoral cooperation is an important factor in preventing and controlling child marriage. The development of cooperation between non-governmental organizations and government organizations in Indonesia is one of these examples (103).

The earthquake of 2003 in Bam caused significant changes in the social, economic, and family systems. The loss of life resulting from the earthquake led to the formation of new family structures, which, in many cases, resulted in divorce. Besides the immediate effects, the earthquake is also likely to have long-term impacts on the future marriages of young girls. Getting rid of loneliness in parents who lost their family in the earthquake is one of the effects. Additionally, the proximity to Afghanistan, one of the largest drug producers and distributors globally (42), has a substantial influence. This influence is dependent on the culture, traditions, and social conditions of the region. The transit and distribution of drugs contribute to an increase in crime and social issues, such as addiction, divorce, and are linked to the breakdown of family foundations and a rise in child marriages.

In addition, advancements in technology such as access to the Internet and virtual networks have led to significant social changes that can impact child marriage. Research indicates that virtual networks can help combat GCM by raising awareness and offering information about the repercussions of early marriage (72, 92). Conversely, exposure to erotic and pornographic content on these platforms may contribute to an increase in early marriage among girls by heightening sexual desire and the risk of unintended pregnancy (104–107).

Other factors affecting GCM include policies and laws in society. In Iran, the increase in the age of marriage, the decrease in the fertility rate, and the aging of the population have led to the revision of demographic policies. Policies such as changing the reproductive age, simultaneous education of married and unmarried girls in school, granting facilities for marriage, and having children are among the policies that affect GCM (36, 108, 109). The announcement of the directive to lower the age of childbearing to 10 years, along with the provision of incentives for couples who marry and have children, encourages society to promote early marriage for girls. Additionally, the interactions of married girls in schools expose unmarried girls to a wealth of information. Discussions about the joys of marriage—such as wedding ceremonies, travel, and receiving gifts, —tempt unmarried girls and contribute to the prevalence of early marriage.

Even though GCM has benefits such as increasing the fertility rate, it also has countless consequences and harms such as increasing the rate of divorce, mental problems, suicide, and female heads of the household (as a result of increased divorce). It seems that decision-making about the continuation, control, and management of GCM requires the implementation of policies that minimize its harm while being beneficial (110). Defects and shortcomings in laws can also affect the spread and continuation of child marriage. Paying attention to the main elements of the GCM law, including the minimum age of marriage and informed and free individual consent, reveals the shortcomings in the law (111). The first requirement of the marriage laws is to determine the minimum age for marriage. While the civil laws of Iran do not explicitly mention the age of marriage, conflicts and inconsistencies can be seen in the current laws, which somehow reduce the deterrent power of the laws. Another aspect of marriage law refers to “informed and free individual consent.” Evidence shows that including exceptions such as parental consent, court recognition, and customary law weakens legal protections against early marriage. For example, if the family consents to GCM due to poverty (112, 113), laws may officially approve a harmful custom. The “growth concept” for issuing marriage licenses in the law also has serious flaws. In Iran, girls under the age of 15 must go to court to obtain a marriage license, where their maturity—specifically their understanding of financial matters—is assessed by a judge. Typically, judges pose a few questions to evaluate the children’s competence in financial management and ownership. If deemed competent, the girls receive a of “certificate maturity,” which permits them to marry at a young age. However, the criteria and assessment methods employed in this process appear to be fundamentally flawed (51).The existence of customary or religious legal systems alongside the civil legal system is another problem that has been mentioned in other studies (87).

### 4.1 Strengths and limitations

This study has several strengths. First, it is one of the few studies that identified the drivers of GCM using the SEM as a conceptual framework. Second, the sampling was conducted with maximum diversity, including participants such as girls married under the age of 15, local policymakers, legislators, clergy, teachers, and husbands. Third, an independent researcher was employed to ensure the accuracy of the survey during the process of generating codes and categories. However, there were limitations in this research. We could not include a diverse sample in terms of Sunni religion. Many legislators declined to participate in the study due to their reluctance to disclose the facts, and only a small number were interviewed. Given that we considered a 15-year time frame for the inclusion of eligible girls, it may be challenging to recall the reasons for marriage in earlier years. However, we adhered to the principle of diversity in sample selection to capture as many reasons for girls’ marriages as possible. Additionally, we recognized that the participation of husbands in the study could influence the girls’ reporting of the reasons for their marriages. To address this concern, we explained the study objectives to both husbands and girls prior to the interviews, and obtaining informed consent helped to mitigate this issue.

## 5 Conclusion

**Figure 1:**
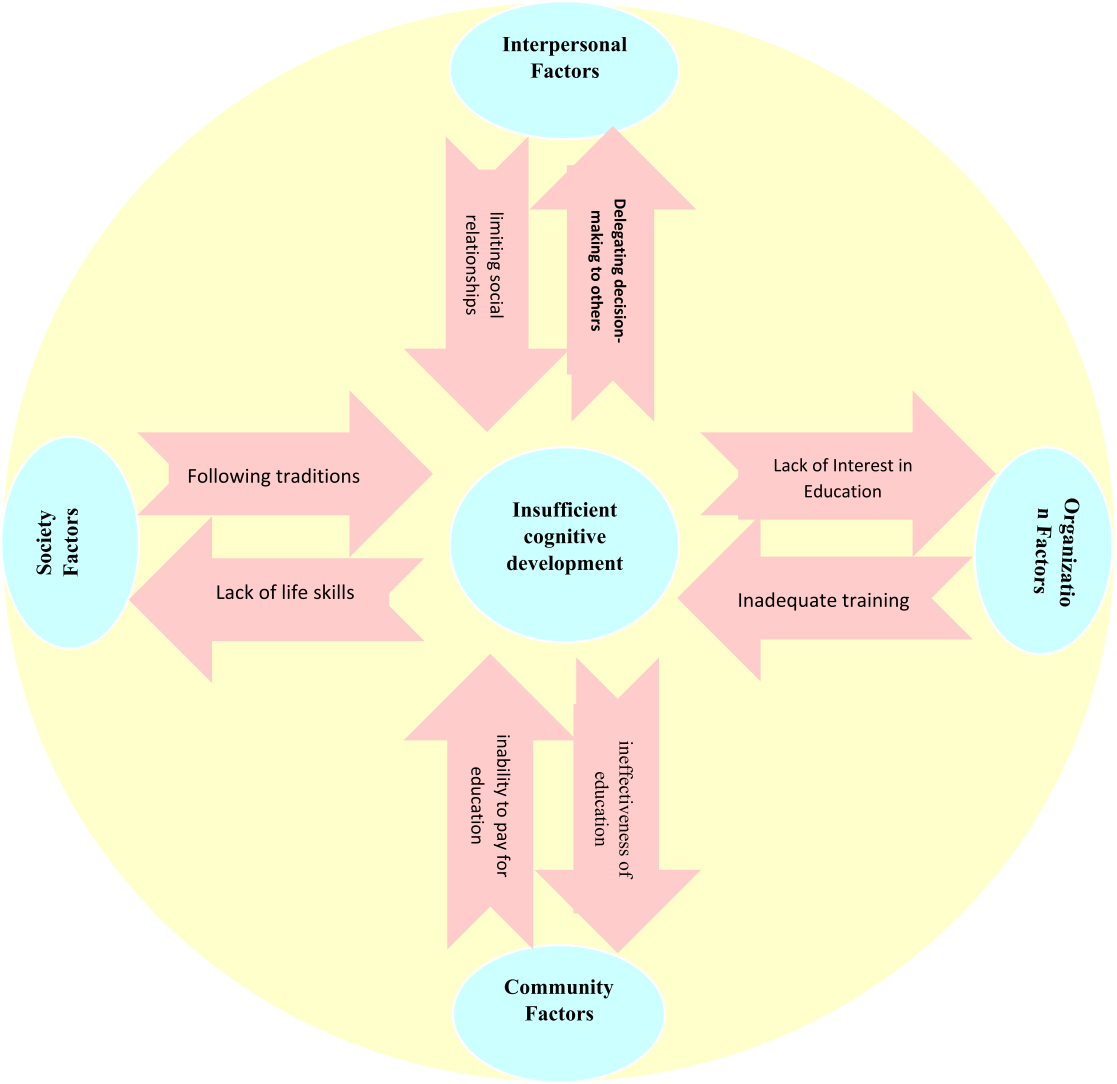
Interrelationships of insufficient inferential cognitive development factors at the level of the socio-ecological model.

The findings indicate that the SEM is a suitable framework for explaining the driving factors of child marriage. To prevent early marriage, the factors that drive it were examined within a set of systems. Similar to systems theory, the relationship between components is analyzed as the connection between a system and other systems. GCM is not viewed as solely a personal or family decision but rather as a result of the interactions among various systems in a society, which can either increase or decrease its prevalence. Girls, family, school, culture, religion, politics, and laws are among the most significant systems influencing child marriage. A thorough analysis of the relationships among these systems and how they impact early marriage can help illuminate its lesser-known aspects and guide policymakers in implementing targeted interventions. Therefore, managing, controlling, and preventing GCM requires comprehensive efforts at the regional, national, and international levels. At the regional and national levels, many shortcomings can be observed, while at the international level, policies are implemented that significantly impact the future of girls. For instance, international economic sanctions in Iran present a significant obstacle to the education and empowerment of women, which has been identified as one of the most crucial factors influencing the decrease in GCM rates (114). The unstable economic conditions within families and the inability to afford education compel girls to abandon schooling and enter marriage at a young age. In Figure 1, we have shown an example of the interrelationships of the driving factors of child marriage. Insufficient heuristic cognitive development at the individual level causes girls to leave the marriage decision to their parents. On the other hand, parents affect their cognitive development by limiting social relationships. Girls, with their disinterest in education, challenge the supply of human resources of the organization, and the organizations neglect the cognitive development of girls by failing to train them. At the social level, girls are left out of the economic cycle of the society by considering education to be ineffective and dropping out. Economic instability leads to families being unable to cover the costs of education, resulting in decreased chances of girls’ education. At the community level, the lack of girls’ life skills affects their social life, and society influences girls’ cognitive development by upholding traditions.

## Data Availability

The original contributions presented in the study are included in the article/supplementary material, further inquiries can be directed to the corresponding author.
