# Supplementary material for: Factors associated with girl child marriage in Iran: a qualitative socio-ecological approach
Source: Front Public Health. 2025 Jan 21;12:1477197. doi: 10.3389/fpubh.2024.1477197 (PMC11790579; doi:10.3389/fpubh.2024.1477197)
Supplement: Supplementary file 1 [file Table_1.docx]

**Table S1**: Standards for Reporting Qualitative Research (SRQR)

| **No.** | **Topic** | | **Item** | **page number** | |
| --- | --- | --- | --- | --- | --- |
| **Title and abstract** | | | | | |
| S1 | Title | | Concise description of the nature and topic of the study Identifying the study as qualitative or indicating the approach (e.g., ethnography,  grounded theory) or data collection methods (e.g., interview, focus  group) is recommended | 1 | |
| S2 | Abstract | | Summary of key elements of the study using the abstract format of  the intended publication; typically includes background, purpose,  methods, results, and conclusions | 2 | |
| **Introduction** | | | | | |
| S3 | Problem formulation | | Description and significance of the problem/phenomenon studied;  review of relevant theory and empirical work; problem statement | 3 | |
| S4 | Purpose or research question | | Purpose of the study and specific objectives or questions | 3,4 | |
| **Methods** | | | | | |
| S5 | Qualitative approach and research paradigm | | Qualitative approach (e.g., ethnography, grounded theory, case study,  phenomenology, narrative research) and guiding theory if appropriate;  identifying the research paradigm (e.g., post positivist, constructivist/  interpretivist) is also recommended; rationaleb | 4 | |
| S6 | Researcher characteristics and reflexivity | | Researchers’ characteristics that may influence the research, including  personal attributes, qualifications/experience, relationship with  participants, assumptions, and/or presuppositions; potential or actual  interaction between researchers’ characteristics and the research  questions, approach, methods, results, and/or transferability | 5 | |
| S7 | Context | | Setting/site and salient contextual factors; rationaleb | 4 | |
| S8 | Sampling strategy | | How and why research participants, documents, or events were  selected; criteria for deciding when no further sampling was necessary  (e.g., sampling saturation); rationaleb | 4-5 | |
| S9 | Ethical issues pertaining to human subjects | | Documentation of approval by an appropriate ethics review board  and participant consent, or explanation for lack thereof; other  confidentiality and data security issues | 5 | |
| S10 | Data collection methods | | Types of data collected; details of data collection procedures including  (as appropriate) start and stop dates of data collection and analysis,  iterative process, triangulation of sources/methods, and modification  of procedures in response to evolving study findings; rationaleb | 4-5 | |
| S11 | Data collection instruments and technologies | | Description of instruments (e.g., interview guides, questionnaires)  and devices (e.g., audio recorders) used for data collection; if/how the  instrument(s) changed over the course of the study | 4-5 | |
| S12 | Units of study | | Number and relevant characteristics of participants, documents, or  events included in the study; level of participation (could be reported  in results) | 4-5 | |
| S13 | Data processing | | Methods for processing data prior to and during analysis, including  transcription, data entry, data management and security, verification  of data integrity, data coding, and anonymization/deidentification of  excerpts | 5 | |
| S14 | Data analysis | | Process by which inferences, themes, etc., were identified and  developed, including the researchers involved in data analysis; usually  references a specific paradigm or approach; rationale b | 5 | |
| S15 | Techniques to enhance trustworthiness | | Techniques to enhance trustworthiness and credibility of data analysis  (e.g., member checking, audit trail, triangulation); rationaleb | 5 | |
| **Results/findings** | | | | | |
| S16 | Synthesis and interpretation | | Main findings (e.g., interpretations, inferences, and themes); might  include development of a theory or model, or integration with prior  research or theory | 6-13 | |
| S17 | Links to empirical data | | Evidence (e.g., quotes, field notes, text excerpts, photographs) to  substantiate analytic findings | Table2 | |
| **Discussion** | |  | | | 13-15 |
| S18 | Integration with prior work, implications, transferability, and contribution(s) to the field | | Short summary of main findings; explanation of how findings  and conclusions connect to, support, elaborate on, or challenge  conclusions of earlier scholarship; discussion of scope of application/  generalizability; identification of unique contribution(s) to scholarship  in a discipline or field | 16 | |
| S19 | Limitations | | Trustworthiness and limitations of findings | 16 | |
| **others** | | | | | |
| S20 | Conflicts of interest | | Potential sources of influence or perceived influence on study conduct and conclusions; how these were managed | 17 | |
| S21 | Funding | | Sources of funding and other support; role of funders in data collection, interpretation, and reporting | 17 | |

**TableS2**. Inclusion and exclusion criteria participants in the study

| **participants** | **Inclusion Criteria** | **Exclusion Criteria** |
| --- | --- | --- |
| **Child Bride** | - Women aged 15-30 - Marriage under the age of 15 - Being Iranian - Native of the region - Consent to participate in the study | - Women who got married under the age of 15 before the earthquake or got married less than 5 years after the earthquake (because to the social effects of the earthquake) - Marriage at the age of more than 15 years - Being non-Iranian - Non-native people of the region - Lack of consent to continue the interview |
| **Family Member** | - Have a daughter with eligible characteristics - Consent to participate in the study | - Lack of consent to continue the interview |
| **Informants** | - Being involved in the issue of child marriage - Consent to participate in the stud | - Lack of consent to continue the interview |
| **Policymaker** | - Having 5 years of work experience - Consent to participate in the stud | - Lack of consent to continue the interview |

**Table S3:** The interview guide for the groups interviewed

| 1. **Guiding questions for eligible girls** |
| --- |
| 1. What is your opinion on marriage? Please explain your thoughts. 2. Do you remember when you believed you were ready for marriage? Please explain your thoughts. 3. What motivated your decision to get married? Please explain your reasoning. 4. Have you ever questioned why you chose to get married at this age? If so, please explain your reasoning. 5. Who were the most influential individuals in your decision to get married, and why? Please explain. 6. What were the opinions of your family and friends regarding your marriage? Please explain why they felt that way. 7. Do you believe that programs on television, radio, and social media platforms (such as Instagram, Telegram, WhatsApp, ETA, Rubika, etc.) have influenced your motivation to get married? Why? Please explain. 8. Do you believe that societal policies and decisions have influenced your choice to get married? Please explain. 9. Do you believe that religious factors played a significant role in your decision to get married? Why? Please explain. 10. Are there any issues within the traditions and culture that promote early marriage? If so, what are they? Please explain. 11. Is early marriage significant in your culture? Why? Please provide an example   If there is a topic you would like to add, please let me know |
| 1. **Guiding questions for husbands** |
| 1. What is your opinion on marriage? Please explain. 2. What criteria did you consider important for marriage? Please explain. 3. What qualities and characteristics do you believe a woman should possess for marriage? Please explain your reasoning. 4. Have you thought about marrying a younger girl? If so, please explain your reasons. 5. Whom did you consult regarding the selection of a wife? Why? Please explain. 6. Who encouraged you to choose a wife under the age of 15? Please explain. 7. Have you ever asked yourself why you married a younger woman? If so, could you explain your reasoning. 8. What was the opinion of your family and friends regarding the marriage of a young girl? Please explain their perspectives. 9. Do you believe that programs on television, radio, and social media platforms (such as Instagram, Telegram, WhatsApp, Ita, Rubika, etc.) have influenced your motivation to get married? Why? Please explain. 10. Do you believe there are any societal policies or decisions that could influence your choice to get married? Please explain. 11. Are there any cultural issues (such as traditions, religion, and values) that promote underage marriage? If so, what are they? Please provide examples.   If there is a topic you would like to add, please let me know |
| 1. **Guide questions for parents** |
| 1. What is your opinion on the girl child marriage? Please explain. 2. Do you remember the first time you discussed marriage with your daughter? Please explain. 3. In your opinion, what factors contribute to girl child marriage in Bam City? Please explain your reasoning. 4. What beliefs and opinions led you to agree to your daughter's marriage? Please explain. 5. Who influenced your decision to allow your daughter to marry? Why? Please explain. 6. In your opinion, what factors contribute to child marriage? Please explain your reasoning. 7. Do you believe that social policies and decisions influence your choice to marry off your daughter? 8. If I have a 15-year-old daughter, what are your thoughts on her getting married? Why? Please explain.   If there is a topic you would like to add, please let me know |
| 1. **Guide questions for informants** |
| 1. What is your opinion on the girl child marriage? Please explain. 2. In your opinion, what factors contribute to child marriage in Bam City? Please explain. 3. What are the most significant ideas and beliefs that contribute to child marriage? Please explain. 4. In your opinion, what factors contribute to the marriage of children? ( students, health center, adolescent) Please explain. 5. What conditions and characteristics do you believe a girl should possess in order to get married? Please explain your reasoning. 6. Do students consult you about marriage? If so, please explain the topics they most frequently discuss. (teacher) 7. 10. In your opinion, how can school influence a student's future marriage? Please explain your reasoning (teacher) 8. What type of care do you offer for adolescent girls? Please explain. (health care provider) 9. Do clients discuss marriage with you? Please explain. (health care provider) 10. Is there a letter and instructions regarding child marriage for schools and health centers? How would you evaluate them? Please explain.   If there is a topic you would like to add, please let me know |
| 1. **Guiding questions for key and local policy makers** |
| 1. What is your opinion on the girl child marriage? Please explain. 2. In your opinion, what factors contribute to child marriage in Bam City? Please explain. 3. What are the most significant ideas and beliefs that contribute to child marriage? Please explain. 4. In your opinion, what factors contribute to child marriage? Please explain. 5. How do you evaluate society's policies regarding the marriage of girls? Please explain. 6. In your opinion, what factors influence the establishment of child marriage laws? How do you assess these rules? Please explain. 7. If you have a daughter under the age of 15, do you support her marriage? What is your reasoning? Please explain.   If there is a topic you would like to add, please let me know. |
